# Seasonality of influenza-like illness and short-term forecasting model in Chongqing from 2010 to 2022

**DOI:** 10.1186/s12879-024-09301-4

**Published:** 2024-04-23

**Authors:** Huayong Chen, Mimi Xiao

**Affiliations:** School of Public Health, Research Center for Medical and Social Development, Chongqing Medical University, 1 Yixueyuan Road, Yuzhong District, 400016 Chongqing, P. R. China

**Keywords:** Influenza-like illnesses (ILI), Time series, Seasonal auto-regressive integrated moving average with eXogenous factors (SARIMAX), Forecast

## Abstract

**Background:**

Influenza-like illness (ILI) imposes a significant burden on patients, employers and society. However, there is no analysis and prediction at the hospital level in Chongqing. We aimed to characterize the seasonality of ILI, examine age heterogeneity in visits, and predict ILI peaks and assess whether they affect hospital operations.

**Methods:**

The multiplicative decomposition model was employed to decompose the trend and seasonality of ILI, and the Seasonal Auto-Regressive Integrated Moving Average with exogenous factors (SARIMAX) model was used for the trend and short-term prediction of ILI. We used Grid Search and Akaike information criterion (AIC) to calibrate and verify the optimal hyperparameters, and verified the residuals of the multiplicative decomposition and SARIMAX model, which are both white noise.

**Results:**

During the 12-year study period, ILI showed a continuous upward trend, peaking in winter (Dec. - Jan.) and a small spike in May-June in the 2–4-year-old high-risk group for severe disease. The mean length of stay (LOS) in ILI peaked around summer (about Aug.), and the LOS in the 0–1 and ≥ 65 years old severely high-risk group was more irregular than the others. We found some anomalies in the predictive analysis of the test set, which were basically consistent with the dynamic zero-COVID policy at the time.

**Conclusion:**

The ILI patient visits showed a clear cyclical and seasonal pattern. ILI prevention and control activities can be conducted seasonally on an annual basis, and age heterogeneity should be considered in the health resource planning. Targeted immunization policies are essential to mitigate potential pandemic threats. The SARIMAX model has good short-term forecasting ability and accuracy. It can help explore the epidemiological characteristics of ILI and provide an early warning and decision-making basis for the allocation of medical resources related to ILI visits.

**Supplementary Information:**

The online version contains supplementary material available at 10.1186/s12879-024-09301-4.

## Background

Influenza is an ongoing public health problem, a simple definition of influenza-like illnesses (ILI) is defined as fever (temperature ≥ 38 °C) with cough or sore throat [1–3]. Each year, influenza epidemics cause a large number of hospitalizations and deaths worldwide, especially among adults over 65 years, placing a significant direct and indirect burden of costs on the health system [4–6].. In China, the burden of influenza-associated influenza-like illness is consistently higher among children (0–14 years), followed by adults (15–59 years) and then older adults (≥ 60 years) [7, 8]. Therefore, predicting the trend and seasonality of ILI has important implications for allocation of related medical resources.

A large number of studies focused on influenza [9–11], influenza virus types A(H1N1), A(H3N2), B, mixed [12–18], COVID-19 [19–24] and other related diseases. The models used include SARIMA, Exponential Smoothing (ETS), Neural Network Autoregressive (NNAR) model, Long-Short Term Memory (LSTM), Self-adaptive AI Model (SAAIM) [25–28], etc. The data used in existing studies were mostly uploaded from influenza surveillance sentinel hospitals into the influenza surveillance system, and most of these data reported cases were confirmed by influenza virus nucleic acid testing. However, in practice when patients seek medical treatment, as long as they show symptoms related to ILI, the resources will be spent on related consultation and treatment. If we only rely on the data collected in the influenza surveillance system, the predicted value from the model might be less than the actual cost of investment. To alleviate this bias, we used the first page data of electronic medical cases uploaded directly by medical institutions, and expanded the search scope of ILI, hence we might be able to obtain a predicted value which would be closer to the actual resources invested by the health system.

## Methods

### Data source

As mentioned earlier, the medical resources will be spent on the treatment of any patients with potential symptoms related to ILI, so the use of only J11 and J12 of the International Classification of Diseases (ICD-10) as the basis for searching, might underestimate the burden of costs on medical institutions. In order to obtain a more accurate estimate of the visitation rates of influenza-like illness in Chongqing, we expanded the scope and selected the relevant codes of ILI in ICD-10 (include J06.900, J10, J11, J12, J15.902, J15.903, J18.900, J18.901, J18.903) as the basis for the search. The data uploaded through the electronic medical record of seven hospitals from January 2010 to May 2022 were collected through the YiDuCloud platform, total number of people included in the study was 1,684,929. These seven hospitals are all large tertiary hospitals in Chongqing, one of which is a children’s hospital, and the scope of medical services basically covers major districts and counties in Chongqing. In this study, YiDuCloud has removed all identifiable patient information, so there is no violation of patient privacy policy. In addition, we collected historical data of monthly average maximum temperature, average minimum temperature, PM2.5 [29] and PM10 from the China Meteorological Administration from 2011 to 2022 to build the model.

### Model establishment

We used the first 90% of the data from January 2010 to May 2022 as the training dataset and the rest as the validation dataset for modeling. And we divided the data into 4 groups (total number of visits, total number of outpatient and emergency departments, total number of inpatients, and average length of stay per month), and subdivided each group into 4 groups (0–1 years old, 2–4 years old, 5–64 years old, ≥ 65 years old) according to the age of high-risk groups of severe cases in Chinese influenza diagnosis and treatment protocol and due to the first year of life is a special period for infants and young children, we made a separate prediction for 0–1 years old. Specifically following steps: stationarity test, optimal model building, residual diagnosis, predictive analysis and evaluation optimal model prediction values. The statistical modeling was analyzed by Stata 17.0 and Python 3.10.

### Model selection

Seasonal Auto-Regressive Integrated Moving Average with eXogenous factors (SARIMAX) was combination of autoregressive (AR) and moving average (MA) models (see appendix materials for AR and MA detailed formulas) and added seasonality with or without differencing.

A Seasonal Auto-Regressive Integrated Moving Average with eXogenous factors ($SARIMAX \left(p,d,q\right)\times \left(P,D,Q\right)s$) model is formed by including additional seasonal terms in the ARIMA models. It is written as follows:

$$\begin{array}{l}\left(1-{\phi }_{1}B-\dots -{\phi }_{p}{B}^{P}\right)\\\left(1-{\varPhi }_{1}{B}^{s}-\dots -{\varPhi }_{P}{B}^{Ps}\right){\left(1-B\right)}^{d}{(1-{B}^{s})}^{D}{y}_{t}\\=c+{\beta }_{t}{X}_{t}+(1+{\theta }_{1}B+\dots +{\theta }_{q}{B}^{q})\\(1+{\varTheta }_{1}{B}^{s}+\dots +{\varTheta }_{Q}{B}^{Qs}){\epsilon }_{t}\end{array}$$

where $$ p$$ is order of the autoregressive part, $$ d$$ is degree of differencing involved, $$ q$$ is order of the moving average part, $$ P$$ is order of the autoregressive seasonality part, $$ D$$ is degree of seasonality differencing involved, $$ Q$$ is order of the moving average seasonality part, $$ s$$ is the length of the seasonal cycle, $$ {X}_{t}$$ is exogenous variable and $$ {\beta }_{t}$$ is parameter.

### Stationarity test

The time series were constructed and combined with Augmented Dickey-Fuller (ADF), Auto Correlation Function (ACF) graphs and Partial Auto Correlation Function (PACF) graphs for identification of smoothness and periodicity of the original time series. If the ADF test *p*-value>0.05, the time series is considered to be non-stationary and we will be difference to eliminate trends and seasonality. The ADF test *p*-value<0.05, the time series can be considered as stationarity, and then the ACF and PACF plots are drawn to select the initial search band of hyperparameters.

### Hyperparameter selection

The initial range of values of $$ p, d, q$$ and $$ P, D, Q, s$$ were determined by the tails and truncations of the ACF and PACF plots. Then the optimal combinations of hyperparameters for the SARIMAX model are determined by matching within the above initial range of values through Grid Search and Akaike information criterion (AIC). Finally, the residual distribution of the optimal model was diagnosed to be consistent with the white noise series, and if so, the model can be used for prediction. We will use the Mean Absolute Percentage Error (MAPE) for the evaluation of the SARIMAX model, the closer the MAPE value is to 0 the better the model is.

## Results

### Stationarity test and time series multiplication decomposition

We conducted ADF test (Appendix Figs. 6, 7, 8, 9 and 10) and time series multiplication decomposition for all groups, and found that a few groups were stationary series, but all groups showed trend and seasonality after multiplication decomposition (Appendix Figs. 1, 2, 3, 4 and 5). Therefore, we performed trend and seasonality difference for all groups, and drew ACF and PACF plots (Appendix Figs. 6, 7, 8, 9 and 10) to determine the scope of Grid Search.

Appendix Fig. 1 shows the multiplication decomposition of all patient visits, in which the number of patient visits in all groups shows an upward trend, with a peak near December to January and a trough around August. The aged 0–1, 2–4 and 5–64 years had a trough near February (Appendix Fig. 1A, B, C), the 2–4 years old had a small peak near May, and the ≥ 65 years old group had a small trough near May (Appendix Fig. 1B, D). In general, the number of visits for ILI were more irregular in the 2–4 age group compared to other groups.

Appendix Fig. 2 shows the time series decomposition of the number of outpatient and emergency department visits. The prevalence trend, peak and trough situation are similar to that of appendix Fig. 1, and the peak and trough difference of the number of 2–4 years old patients due to ILI is the largest (appendix Fig. 2B). Appendix Fig. 3 shows the time series decomposition of the number of inpatients, due to the business adjustment of the YiDuClould platform in 2018, the time series chart has experienced large fluctuations. The peak-valley difference in the number of inpatients aged 2–4 is the largest (appendix Fig. 3B).

Appendix Fig. 4 shows the time series decomposition of the average length of hospitalization per month. LOS in the 0-1-year-old group was the most irregular, with peaks in April, August and November, and low points in January, June and September (Appendix Fig. 4A). In the 2–4 age group, LOS showed a peak in August, a small peak in April, and a trough in June and October (Appendix Fig. 4B). LOS peaks for aged 5–64 occur in February and August, troughs occur in January and June, and there are smaller troughs in October (Appendix Fig. 4C). LOS aged 65 and older peaks in July, with a persistent small peak around March, and troughs in January and September (Appendix Fig. 4D).

Appendix Fig. 5 shows the multiplication decomposition of all patient visits by medical visit type and LOS, which shows that the number of visits is increasing year by year (appendix Fig. 5A). After the outbreak of COVID-19 in 2020, the average LOS per month in ILI (excluding COVID-19 cases) increased significantly (appendix Fig. 5D). However, it is interesting to note that the average LOS per month is the opposite of the seasonal performance of the number of hospitalizations, with the average length of hospitalization per month reaching a low point around January and a peak around August (Appendix Fig. 5C, D).

### Forecast performance of SARIMAX

We determined the initial search range of hyperparameters based on the plotted ACF and PACF graphs, and used grid search and Akaike information criterion (AIC) to determine the best SARIMAX models (Appendix Table 1). We then performed a residual diagnosis on the residuals of the optimal model, which showed that the residuals for all groups were white noise (Appendix Figs. 11, 12, 13, 14 and 15), indicating that we could use these models for the following predictions.

**Fig. 1:**
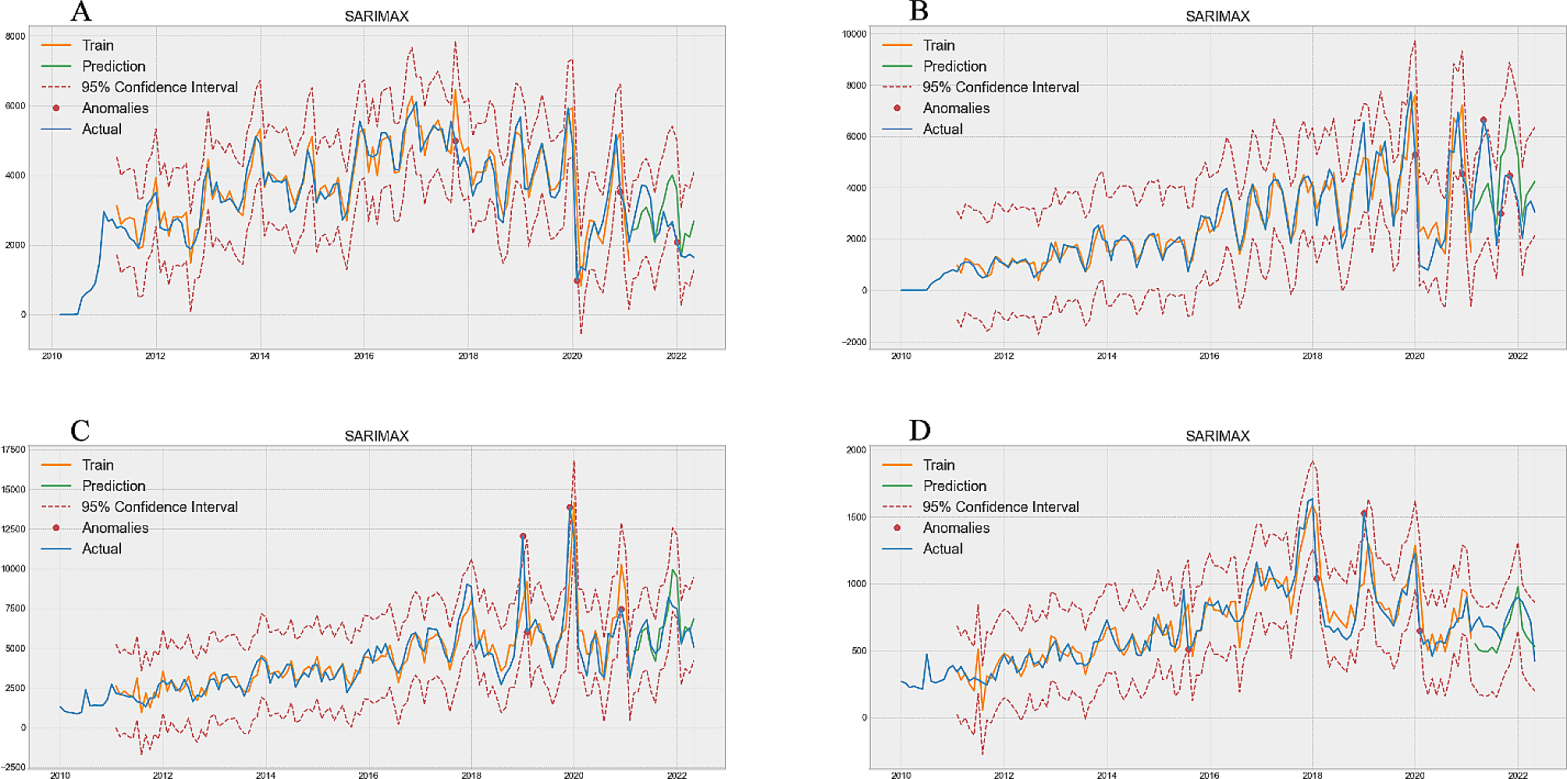
Optimal SARIMAX model prediction of number of patients. **A** is 0–1 years old group, **B** is 2–4 years old group, **C** is 5–64 years old group, **D** is ≥ 65 years old group

Figure 1 predicts the total number of visits. Figure 1A, B, C, and D correspond to projections for 0–1 years old group (Train MAPE = 0.1166, Test MAPE = 0.2850, Table 1), 2–4 years old group (Train MAPE = 0.2062, Test MAPE = 0.3635, Table 1), 5–64 years old group (Train MAPE = 0.1593, Test MAPE = 0.1248, Table 1), and ≥ 65 years old group (Train MAPE = 0.1376, Test MAPE = 0.1867, Table 1), respectively. The 5–64 age group performed best, with a difference of 0.0345 (Table 1) MAPE between the training and the test set, and the 0–1 years old group had the worst performance, with a difference of 0.1684 (Table 1) in MAPE value. The red dots in the figure represent the true value that exceeds the 95% Confidence Interval predicted by the SARIMAX model, and the outliers that exceed the upper limit are our main objects of concern. One abnormal phenomenon can be found in the test set in the 2–4 age group, that exceeded the upper limit of our 95% prediction interval (Fig. 1B). Figure 1 predicts the total number of visits. Figure 1A, B, C, and D correspond to projections for 0–1 years old group (Train MAPE = 0.1166, Test MAPE = 0.2850, Table 1), 2–4 years old group (Train MAPE = 0.2062, Test MAPE = 0.3635, Table 1), 5–64 years old group (Train MAPE = 0.1593, Test MAPE = 0.1248, Table 1), and ≥ 65 years old group (Train MAPE = 0.1376, Test MAPE = 0.1867, Table 1), respectively. The 5–64 age group performed best, with a difference of 0.0345 (Table 1) MAPE between the training and the test set, and the 0–1 years old group had the worst performance, with a difference of 0.1684 (Table 1) in MAPE value. The red dots in the figure represent the true value that exceeds the 95% Confidence Interval predicted by the SARIMAX model, and the outliers that exceed the upper limit are our main objects of concern. One abnormal phenomenon can be found in the test set in the 2–4 age group, that exceeded the upper limit of our 95% prediction interval (Fig. 1B).

**Table 1:** Optimal SARIMAX Models’ Mean Absolute Percentage Error (MAPE)

	All Number of Patients	Outpatient and Emergency	Inpatient	Average Length of Stay Per Month
Train				
All Age	0.1271	0.1452	0.1004	0.0417
0–1 years old group	0.1166	0.1565	0.1623	0.0450
2–4 years old group	0.2062	0.2356	0.2933	0.0779
5–64 years old group	0.1593	0.1748	0.1151	0.0738
≥ 65 years old group	0.1376	0.1494	0.2000	0.1848
Test				
All Age	0.1903	0.1889	0.1680	0.1301
0–1 years old group	0.2850	0.3433	0.6944	0.1908
2–4 years old group	0.3635	0.3403	0.3880	0.0800
5–64 years old group	0.1248	0.1288	0.1444	0.1787
≥ 65 years old group	0.1867	0.1730	0.1540	0.1513

**Fig. 2:**
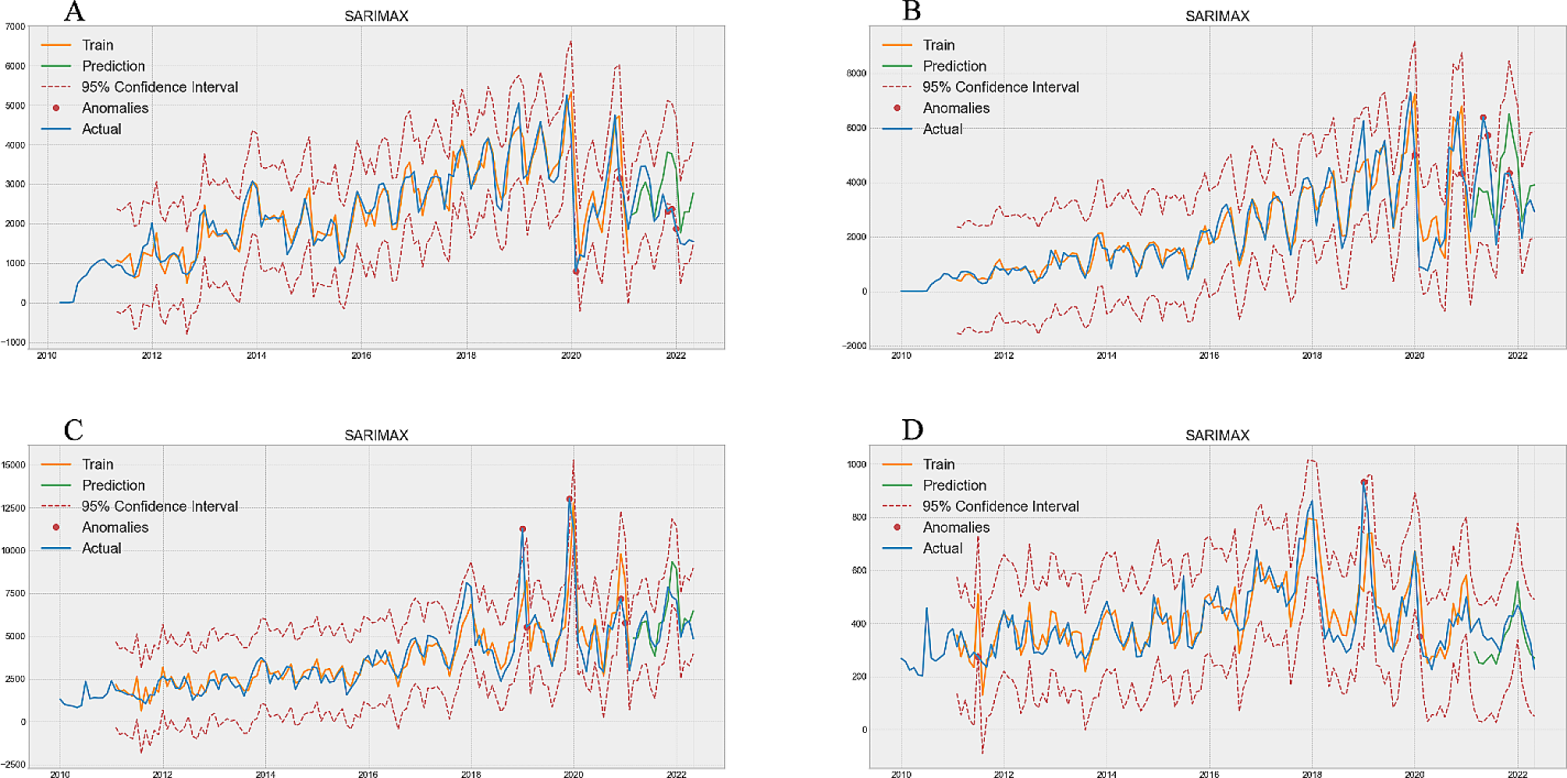
Optimal SARIMAX model prediction of outpatient and emergency. **A** is 0–1 years old group, **B** is 2–4 years old group, **C** is 5–64 years old group, **D** is ≥ 65 years old group

Figure 2 shows the prediction of outpatient and emergency department attendance. Figure 2A, B, C, and D correspond to the predictions of the groups aged 0–1 years group (Train MAPE = 0.1565, Test MAPE = 0.3433, Table 1), 2–4 years group (Train MAPE = 0.2356, Test MAPE = 0.3403, Table 1), 5–64 years group (Train MAPE = 0.1748, Test MAPE = 0.1288, Table 1), and ≥ 65 years group (Train MAPE = 0.1494, Test MAPE = 0.1730, Table 1), respectively. Among them, the ≥ 65 age group has the best performance, and the MAPE difference between the training set and the test set is 0.0236, and the 0–1 years old group had the worst performance, with a difference of 0.1868 in MAPE value (Table 1). In the test set, the true value of outpatient and emergency department attendance exceeded the 95%CI upper limit in two cases (Fig. 2B).

**Fig. 3:**
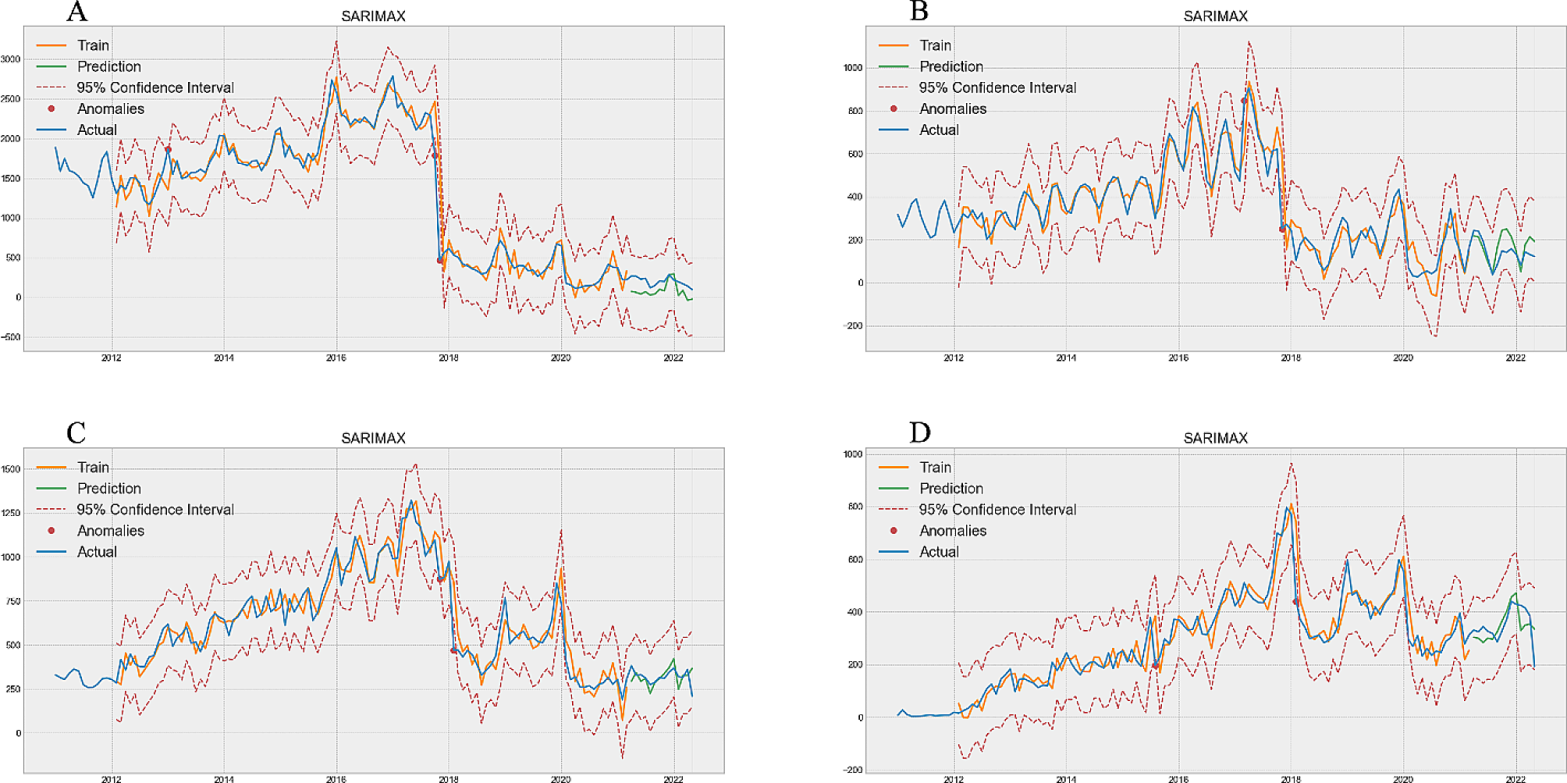
Optimal SARIMAX model prediction of inpatient. **A** is 0–1 years old group, **B** is 2–4 years old group, **C** is 5–64 years old group, **D** is ≥ 65 years old group

Figure 3 shows the projected number of inpatients, Fig. 3A, B, C, and D correspond to predictions of 0–1 years old group (Train MAPE = 0.1623, Test MAPE = 0.6944, Table 1), 2–4 years old group (Train MAPE = 0.2933, Test MAPE = 0.3880, Table 1), 5–64 years old group (Train MAPE = 0.1151, Test MAPE = 0.1444, Table 1), and ≥ 65 years old group (Train MAPE = 0.2000, Test MAPE = 0.1540, Table 1), respectively. Among them, the 5-64-year-old group had the best performance, and the MAPE difference between the training set and the test set was only 0.0293 and the 0–1 years old group had the worst performance, with a difference of 0.5321 in MAPE value (Table 1).

**Fig. 4:**
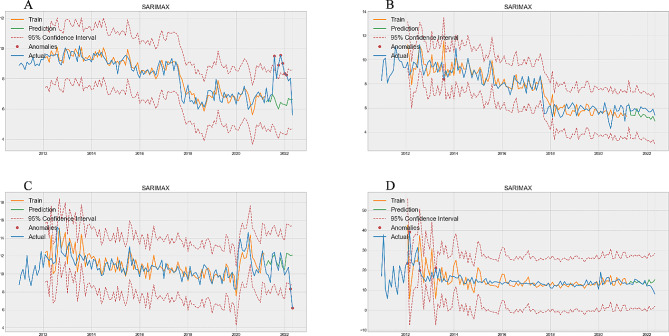
Optimal SARIMAX model prediction of average length of stay per month. **A** is 0–1 years old group, **B** is 2–4 years old group, **C** is 5–64 years old group, **D** is ≥ 65 years old group

Figure 4 shows the prediction of the average LOS per month for the total population. Figure 4A, B, C, and D correspond to the predictions of the four groups aged 0–1 years group (Train MAPE = 0.0450, Test MAPE = 0.1908, Table 1), 2–4 years group (Train MAPE = 0.0779, Test MAPE = 0.0800, Table 1), 5–64 years group (Train MAPE = 0.0738, Test MAPE = 0.1787, Table 1), and ≥ 65 years group (Train MAPE = 0.1848, Test MAPE = 0.1513, Table 1), respectively. Among them, the 2–4 age group has the best performance, and the MAPE difference between the training set and the test set is only 0.0021 and the 0–1 years old group had the worst performance, with a difference of 0.1458 in MAPE value (Table 1). In the test set, the actual average LOS per month in the 0–1 age group exceeded the upper limit 6 times.

**Fig. 5:**
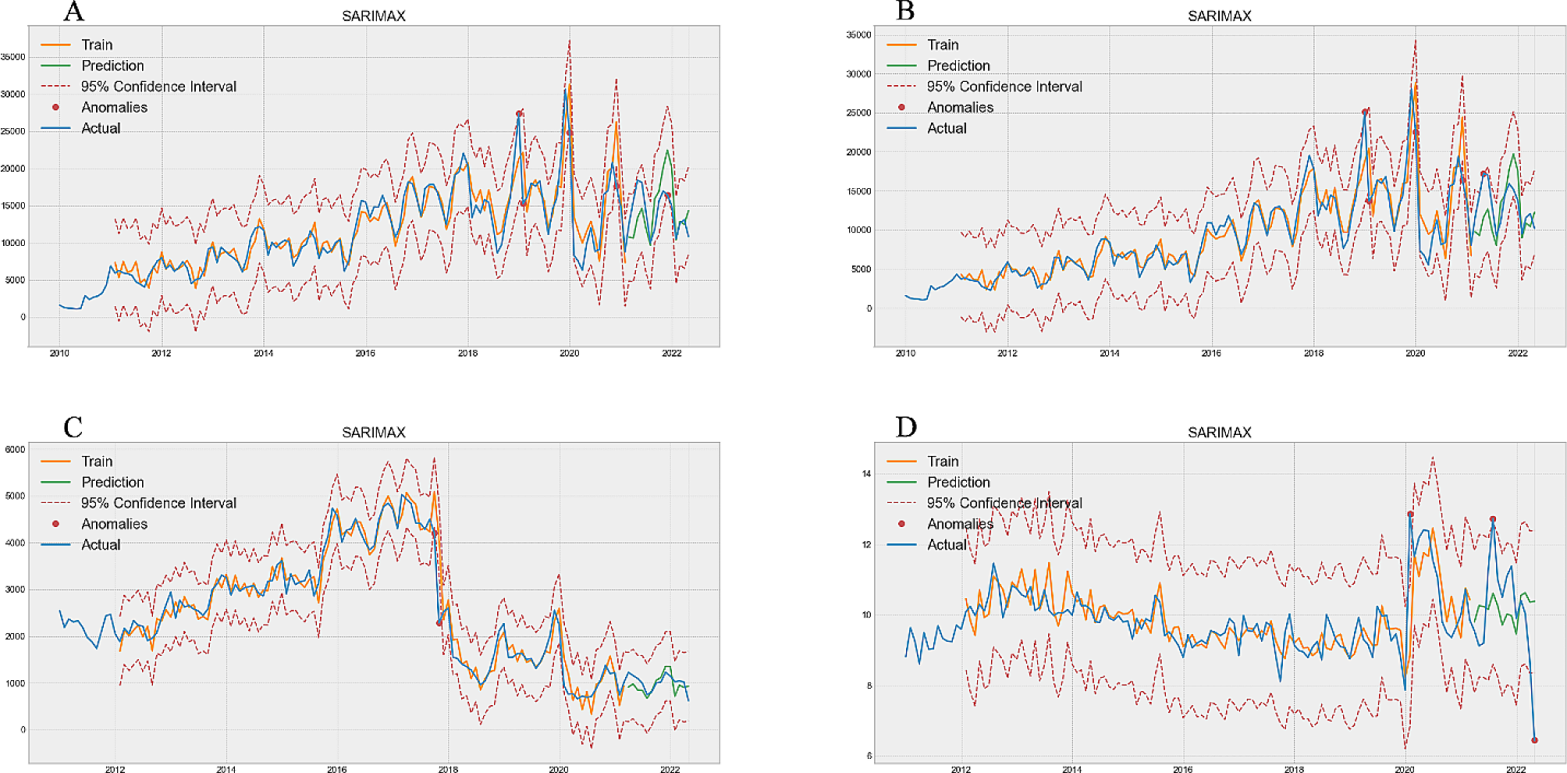
Optimal SARIMAX model prediction of number of patients by medical visit type and LOS. **A** is all patients group, **B** is outpatient and emergency group, **C** is inpatient group, **D** is average length of stay per month

Figure 5 predicts the total number of visits by medical visit type and LOS. Figure 5A predicts the total number of visits (Train MAPE = 0.1271, Test MAPE = 0.1903, Table 1), B is outpatient and emergency group (Train MAPE = 0.1452, Test MAPE = 0.1889, Table 1), C is inpatient group (Train MAPE = 0.1004, Test MAPE = 0.1680, Table 1), D is average LOS per month (Train MAPE = 0.0417, Test MAPE = 0.1301, Table 1). There are two true values exceed 95%CI.

## Discussion

ILI is a year-round disease burden that causes varying degrees of illness, sometimes leading to hospitalization and death [30]. However, current time series surveys and projections for ILI in Chongqing are using the national influenza surveillance system [9, 17, 18, 31–33] and are likely to underestimate ILI’s burden on hospital operations. Therefore, in this study, we applied the SARIMAX time series method, which can effectively capture the cyclical and seasonal changes of diseases [34, 35], to the personal electronic medical data stored in hospitals for many years, to check the prevalence time and intensity of ILI in Chongqing, so as to predict the medical resources needed by the actual treatment of ILI in hospitals. In our study, using ILI data over a 12-year period, we identified an annual seasonal pattern in Chongqing, with influenza activity peaking around December to January each winter, the significant seasonality and periodicity is consistent with the previous studies [9, 10]. The seasonal characteristics of Chongqing are similar to those of Shenyang, but different from Shenzhen, whose ILI(%) peak occurs in summer [36]. In addition, to facilitate early warning, we added abnormal feedback over 95% forecast Confidence Intervals to forecast ILI trends and seasonality. Once the actual number of patients exceeds the 95%CI predicted by the number of ILI patients and the LOS, we believe that ILI begins to affect the normal operation of the hospital and may form an ILI pandemic. At this time, the hospital and the government should make emergency plans in advance according to the actual situation, and allocate the medical materials correspondingly.

We also analyzed the heterogeneity of visits and LOS in ILI, with similar peaks for outpatient and emergency department visits and hospitalizations, but a small spike in April-June in the 0–4-year-old high-risk group of severely ill people, which increases the possibility of influenza virus transmission in institutions such as kindergartens. In the heterogeneity analysis of LOS decomposition, we found that LOS and the number of visits peak at different times, LOS peak in summer, which may indicate that ILI symptoms are more severe in summer, therefore require longer hospitalization time. Moreover, the outbreak of COVID-19 in 2020 significantly affected the treatment of ILI patients and increased the LOS of ILI patients. And LOS in the severely high-risk group is more irregular than that in the normal group, especially in the 0–1 age group, which significantly increased during the epidemic rebound in 2021, indicating that the COVID-19 epidemic had a more serious impact on this group. The possible reason is that the high-risk group may lack prior exposure to the virus and have poor immunity [18], resulting in more unstable disease, and LOS becoming more irregular. The above may also be one of the reasons why the difference in MAPE between the 0–1 years old test set and the training set is larger than the other groups. When LOS of high-risk patients is found to exceed 95%CI of our predictive model, doctors should pay more attention to this group of patients to avoid exacerbation of their disease. These age group differences in ILI seasonality have implications not only for vaccination timing, but also for vaccine composition. Therefore, age heterogeneity may be an important consideration in the future development of immunization policies in Chongqing (for example, one additional vaccination in April-June for severely ill high-risk groups, especially those aged 0–4 years), and may be a useful assessment and reference for other regions with similar climates to Chongqing.

This study also has some limitations. First, we collected data through May 2022, when China’s non-pharmaceutical measures for COVID-19 are still dynamic zero-COVID policy, we did not include China’s dynamic zero COVID-19 policy at that time in the model, resulting in a difference in the MAPE of the training set and the test set, this may increase the error rate in predicting the number of ILI visits in China after full lifting, too. Second, we did not include the COVID-19 attendance data in this prediction model due to its sensitivity, ordinary people lack expertise in COVID-19 diagnosis and may be classified as COVID-19 diagnosis and excluded by us after they have utilized ILI’s treatment resources, which may lead to slight differences between our prediction and the actual situation. The two points above may also be the reason why the predicted values of some test sets of our model differ greatly from the actual values. Longer-term time series will help improve the ability to detect and validate ILI prevalence trends and seasonality. Given that granular temporal surveillance data are often not available when ILI is prevalent, we pooled the data from all analyses to monthly levels, which may hide the variability that can occur at weekly levels, but previous studies have found that such data is not essential for capturing temporal disease transmission patterns [37]. In the follow-up study, we will consider the corresponding actual situation and collect the data to the week for prediction when the data is feasible. If practicable, we can consider including the dynamic zero COVID-19 policy into external variables for model correction, and extend the time of data inclusion to 2024 to enhance the accuracy and applicability of the forecast, so as to achieve the goal of providing reference for public health decision-making in a timely manner. Finally, the service of the seven hospitals included in the YiDuCloud platform might not cover all the Chongqing, so the prediction model is only applicable to the number of ILI visits in the service area of the hospitals above, hence one should be cautious when applying the interpretation to the whole municipal area of Chongqing.

## Conclusion

Despite some limitations, our study provides a strong quantitative estimate of ILI prediction and early warning at the hospital level in Chongqing over a 12-year period. Our results showed that ILI prevalence had a strong seasonality, and the LOS in the critically ill high-risk group was irregular. There was a small peak of the number of patients in the critically ill high-risk group aged 2–4 years from April to June. Because early detection is essential to prevent and control the spread of ILI, our study could be useful for early detection of ILI epidemics or for strengthening surveillance of infection in key populations during periods of high ILI transmission. Therefore, our research results are of great significance for decision-makers to grasp the epidemic trend and seasonality of ILI in time. Prediction analysis based on SARIMAX model is helpful to effectively save medical resources, reduce the burden of medical institutions and health systems, and reduce social and economic costs.

### Electronic supplementary material

Below is the link to the electronic supplementary material.

Supplementary Material 1

## Data Availability

The datasets generated and/or analysed during the current study are not publicly available due YiDuCloud platform requires individuals to use it after registering on the YiDuCloud platform but are available from the corresponding author on reasonable request.

## Abbreviations

ILI: Influenza like illness
SARIMAX: Seasonal Auto Regressive Integrated Moving Average with eXogenous factors
LOS: Length of stay
ICD-10: International Classification of Diseases
ADF: Augmented Dickey-Fuller
ACF: Auto Correlation Function
PACF: Partial Auto Correlation Function
MAPE: Mean Absolute Percentage Error
