# Supplementary material for: Seasonality of influenza-like illness and short-term forecasting model in Chongqing from 2010 to 2022
Source: BMC Infect Dis. 2024 Apr 23;24:432. doi: 10.1186/s12879-024-09301-4 (PMC11036656; doi:10.1186/s12879-024-09301-4)
Supplement: Supplementary file 1 — Supplementary Material 1 [file 12879_2024_9301_MOESM1_ESM.docx]

AR and MA detailed formulas:

An autoregressive model of order $p$ is specified as below:

$$y_{t}=\varphi_{0}+\varphi_{1}y_{t-1}+\varphi_{2}y_{t-2}+\ldots+\varphi_{p}y_{t-p}+\varepsilon_{t}$$

where the lagged value of $y_{t}$ is the predictors, $y_{t-1}, y_{t-2},\ldots y_{t-p}$ are stationaries,$\varphi_{1},\cdot\cdot\cdot,\varphi_{p}$ are parameters, $\varepsilon_{t}$ is white noise series with mean zero

A moving average model of order $q$ is presented as below:

$$y_{t}=\varepsilon_{t}-\theta_{1}\varepsilon_{t-1}-\theta_{2}\varepsilon_{t-2}-\ldots-\theta_{q}\varepsilon_{t-q}$$

where $y_{t}$ as a weighted moving average of the past few forecast errors, $\theta_{1},\cdot\cdot\cdot,\theta_{q}$ are parameters, $\varepsilon_{t},\varepsilon_{t-1},\varepsilon_{t-2},\varepsilon_{t-q}$ are white noise series with mean zero.

The differenced series can be written as follow:

$$y_{t}^{'}=y_{t}-y_{t-1}$$

Where $y_{t}^{'}$ is the change between consecutive observations in the original series.

The Auto-Regressive Integrated Moving Average can be written as

$$y_{t}^{'}=c+\varphi_{1}y_{t-1}^{'}+\ldots+\varphi_{p}y_{t-p}^{'}+\theta_{1}\varepsilon_{t-1}+\ldots+\theta_{q}\varepsilon_{t-q}+\varepsilon_{t}$$

Where $y_{t}^{'}$ is the differenced series, the right-hand side of equation includes both lagged values of $y_{t}$ and lagged errors. This is an $ARIMA(p,d,q)$ model, where $p$ is order of the autoregressive part, $d$ is degree of differencing involved, $q$ is order of the moving average part.

$ARIMA(p,d,q)$ model can be written in backshift notation as below:

$$\left( 1-\varphi_{1}B-\ldots-\varphi_{p}B^{P} \right)\left( 1-B \right)^{d}y_{t}=c+(1+\theta_{1}B+\ldots+\theta_{q}B^{q})\varepsilon_{t}$$

| Appendix Table 1. Optimal SARIMAX Models | | | | |
| --- | --- | --- | --- | --- |
|  | All Number of Patients | Outpatient and Emergency | Inpatient | Average Length of Stay Per Month |
| All Age | SARIMAX(1, 1, 2)x(0, 1, 1, 12) | SARIMAX(1, 1, 2)x(0, 1, 1, 12) | SARIMAX(2, 1, 2)x(1, 1, 2, 12) | SARIMAX(1, 1, 1)x(2, 1, 1, 12) |
| 0-1 years old group | SARIMAX(0, 1, 4)x(0, 1, 1, 12) | SARIMAX(0, 1, 4)x(0, 1, 1, 12) | SARIMAX(0, 1, 3)x(0, 1, 1, 12) | SARIMAX(0, 1, 2)x(0, 1, 2, 12) |
| 2-4 years old group | SARIMAX(2, 1, 1)x(0, 1, 1, 12) | SARIMAX(0, 1, 4)x(0, 1, 1, 12) | SARIMAX(2, 1, 2)x(0, 1, 1, 12) | SARIMAX(1, 1, 2)x(0, 1, 1, 12) |
| 5-64 years old group | SARIMAX(1, 1, 2)x(0, 1, 1, 12) | SARIMAX(0, 1, 3)x(0, 1, 1, 12) | SARIMAX(3, 1, 4)x(1, 1, 1, 12) | SARIMAX(0, 1, 2)x(0, 1, 1, 12) |
| ≥65 years old group | SARIMAX(2, 1, 1)x(0, 1, 1, 12) | SARIMAX(1, 1, 1)x(0, 1, 1, 12) | SARIMAX(0, 1, 0)x(0, 1, 1, 12) | SARIMAX(4, 1, 2)x(1, 1, 1, 12) |


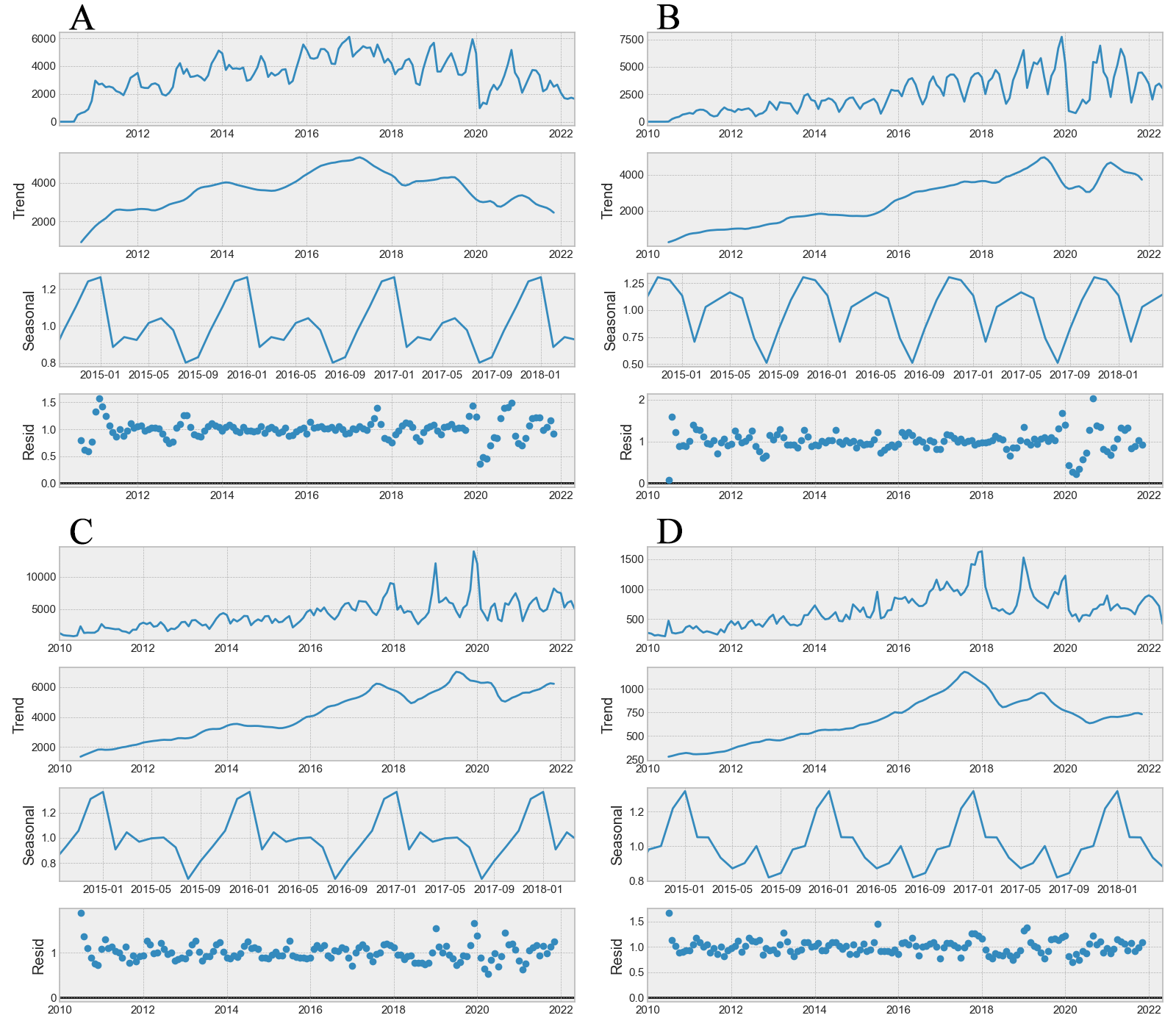
Appendix Figure 1. Time series multiplicative decomposition of number of patients. A is 0-1 years old group, B is 2-4 years old group, C is 5-64 years old group, D is ≥65 years old group.


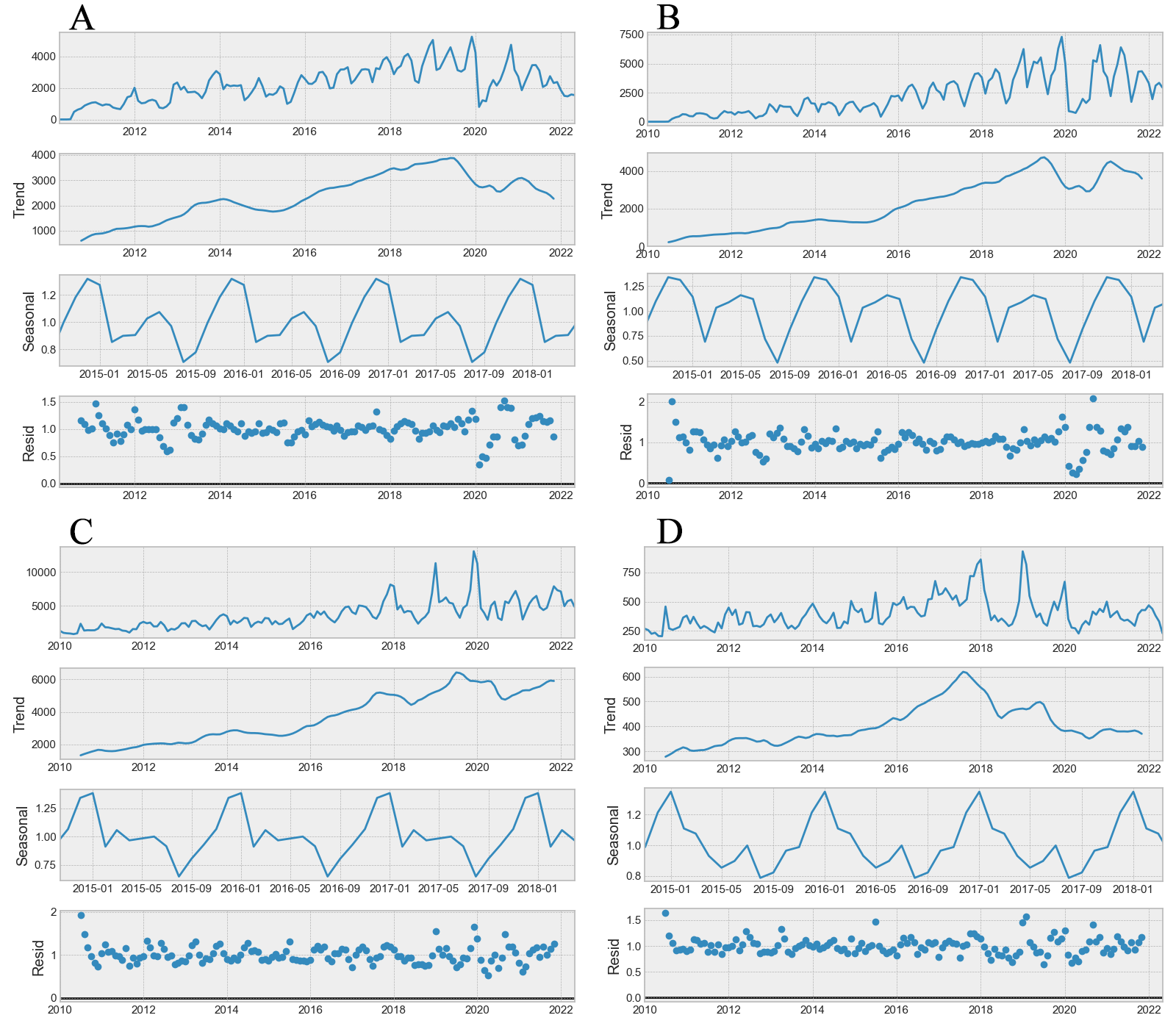


Appendix Figure 2. Time series multiplicative decomposition of outpatient and emergency. A is 0-1 years old group, B is 2-4 years old group, C is 5-64 years old group, D is ≥65 years old group.


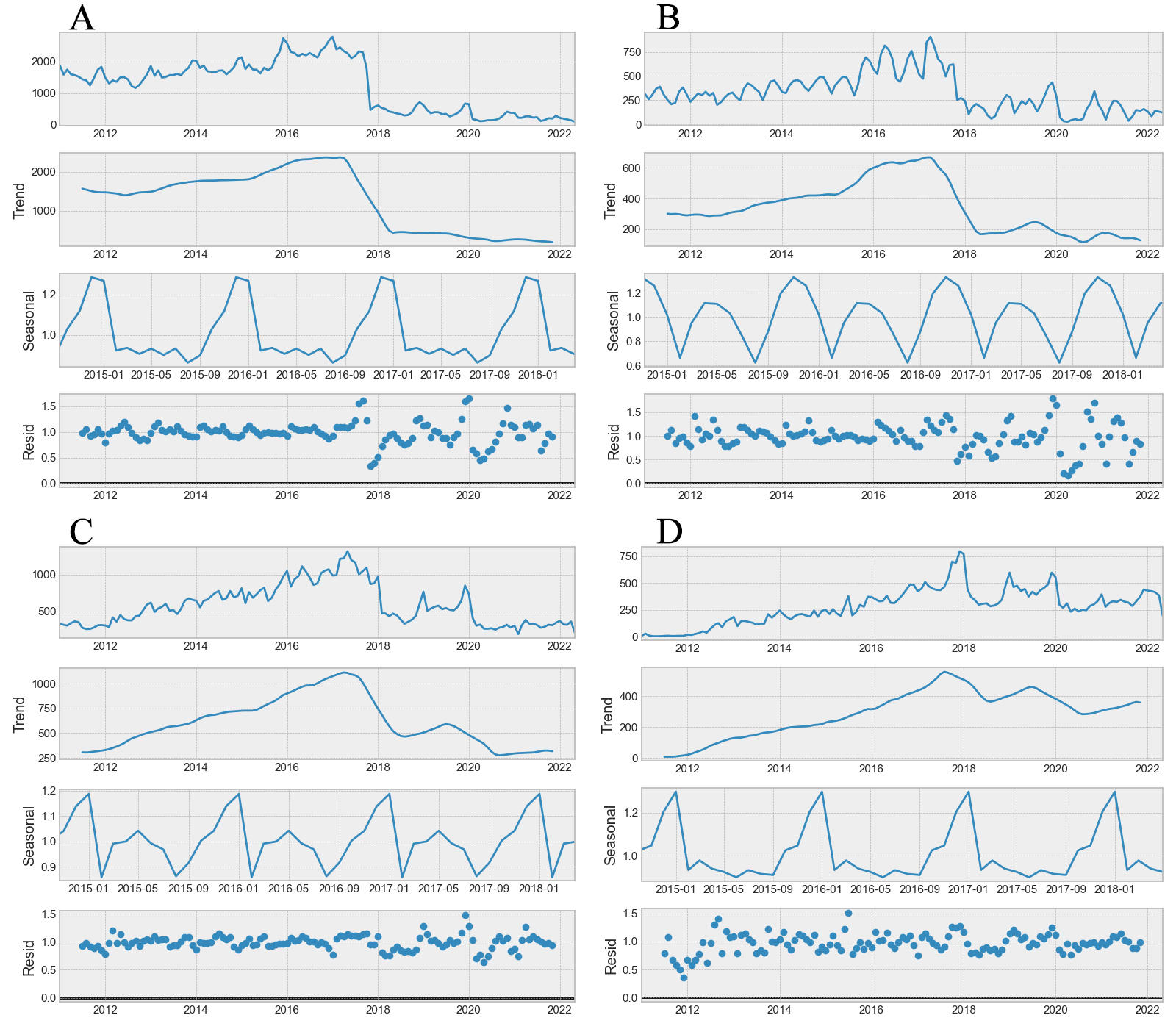


Appendix Figure 3. Time series multiplicative decomposition of inpatient. A is 0-1 years old group, B is 2-4 years old group, C is 5-64 years old group, D is ≥65 years old group.


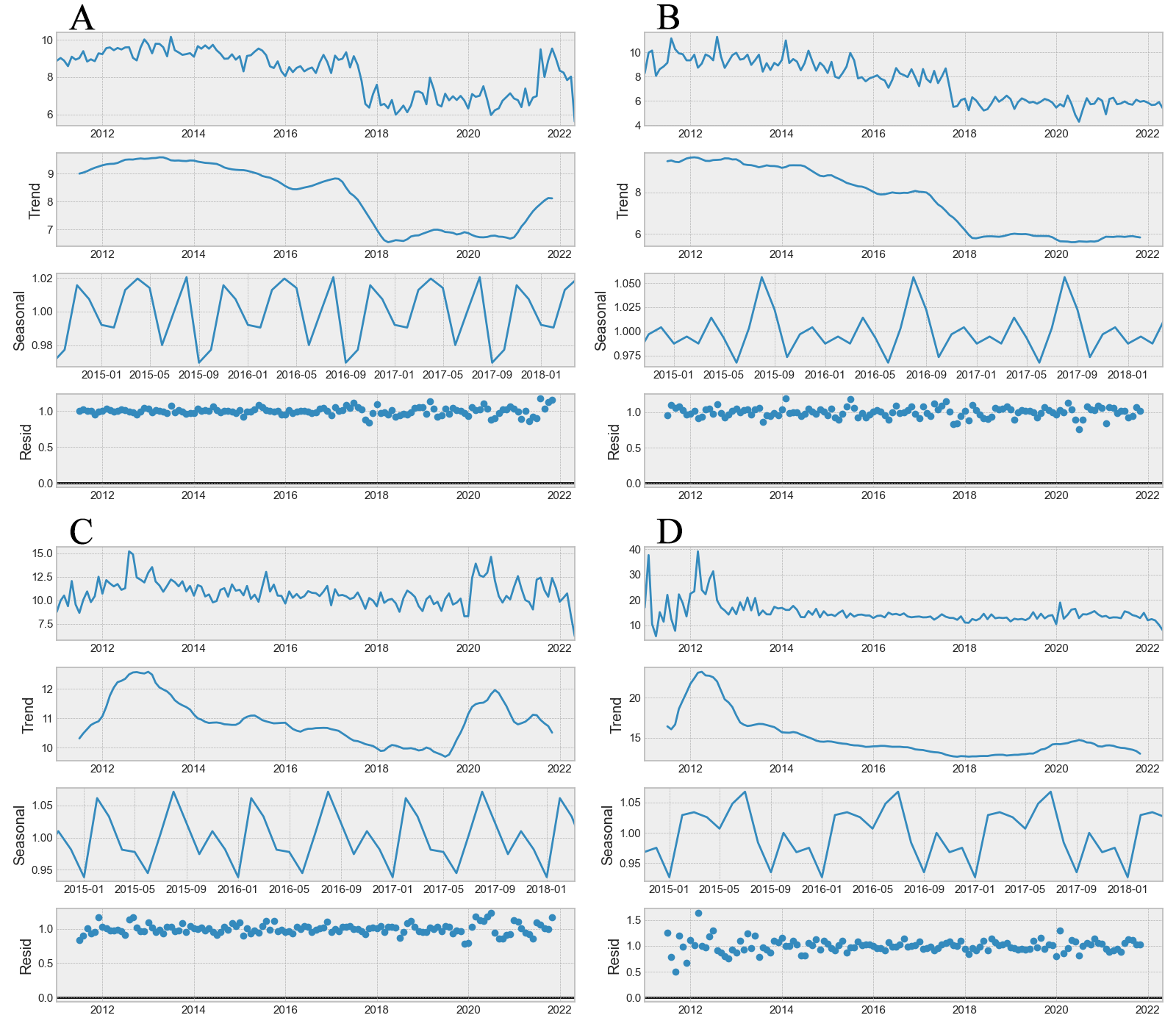


Appendix Figure 4. Time series multiplicative decomposition of average length of stay per month. A is 0-1 years old group, B is 2-4 years old group, C is 5-64 years old group, D is ≥65 years old group.


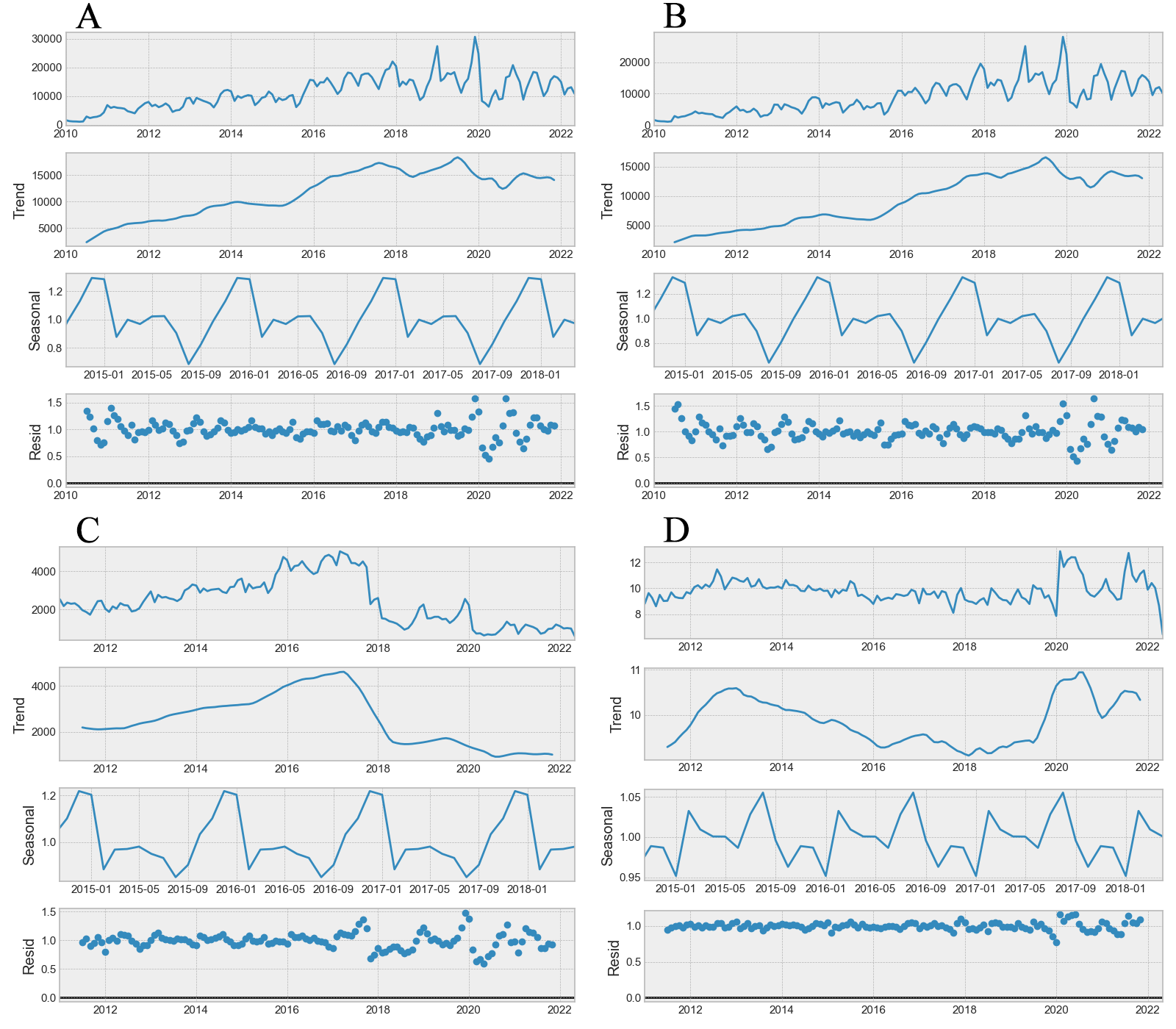


Appendix Figure 5. Time series multiplicative decomposition of number of patients by medical visit type and LOS. A is all patients group, B is outpatient and emergency group, C is inpatient group, D is average length of stay per month.


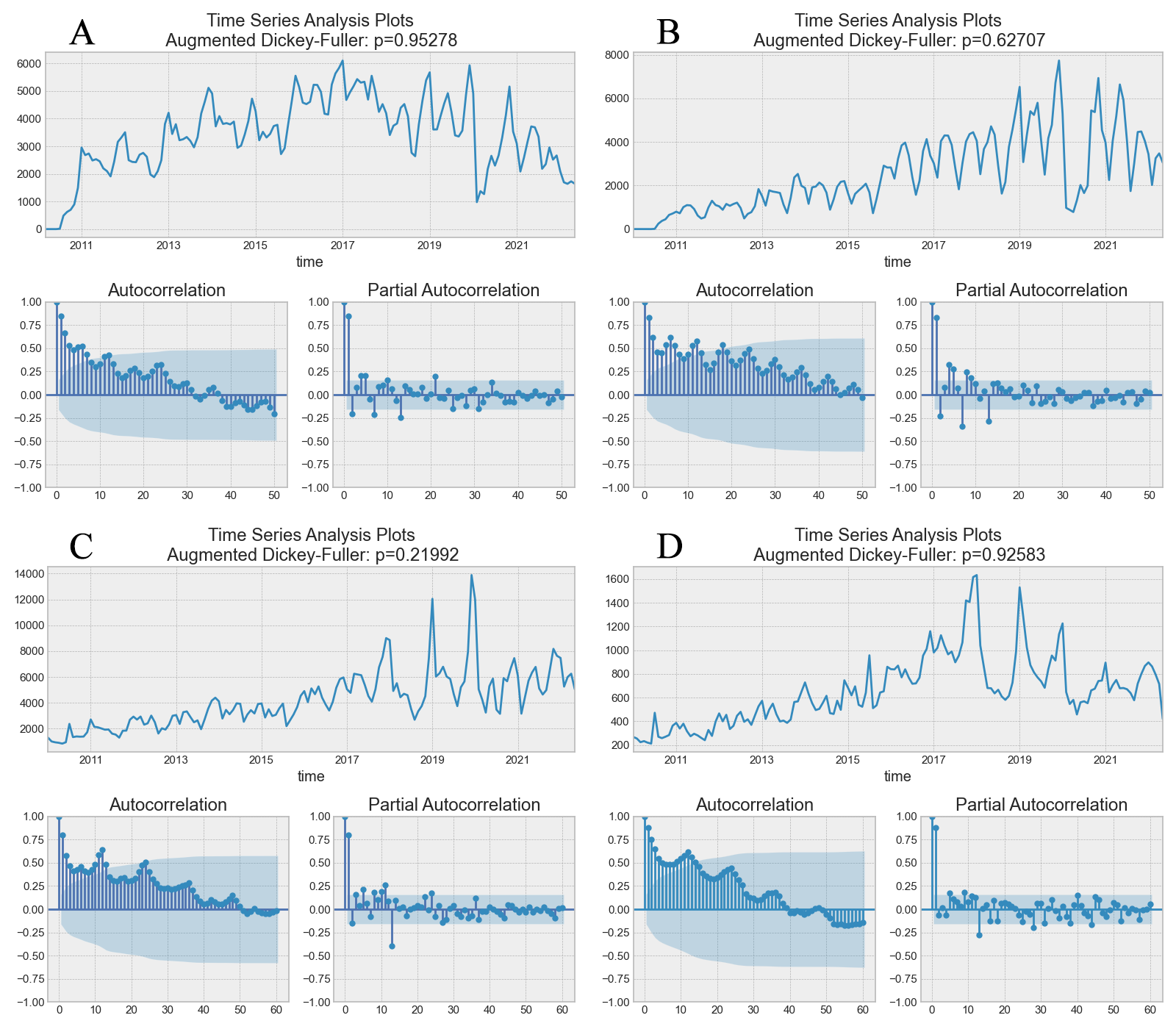


Appendix Figure 6. Augmented Dickey-Fuller test , Auto Correlation Function (ACF) graphs and Partial Auto Correlation Function (PACF) graphs of number of patients. A is 0-1 years old group, B is 2-4 years old group, C is 5-64 years old group, D is ≥65 years old group.


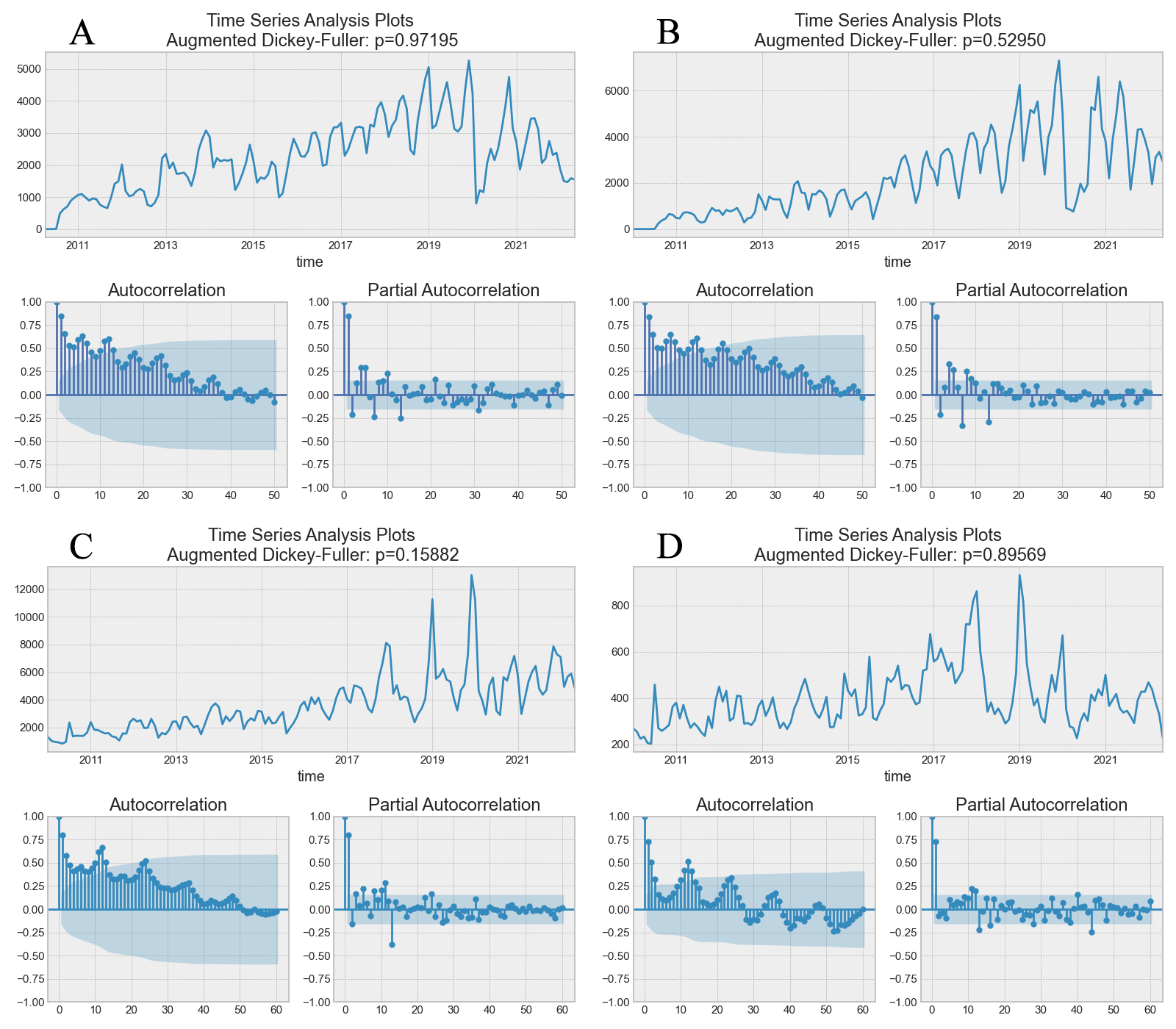


Appendix Figure 7. Augmented Dickey-Fuller test , Auto Correlation Function (ACF) graphs and Partial Auto Correlation Function (PACF) graphs of outpatient and emergency. A is 0-1 years old group, B is 2-4 years old group, C is 5-64 years old group, D is ≥65 years old group.


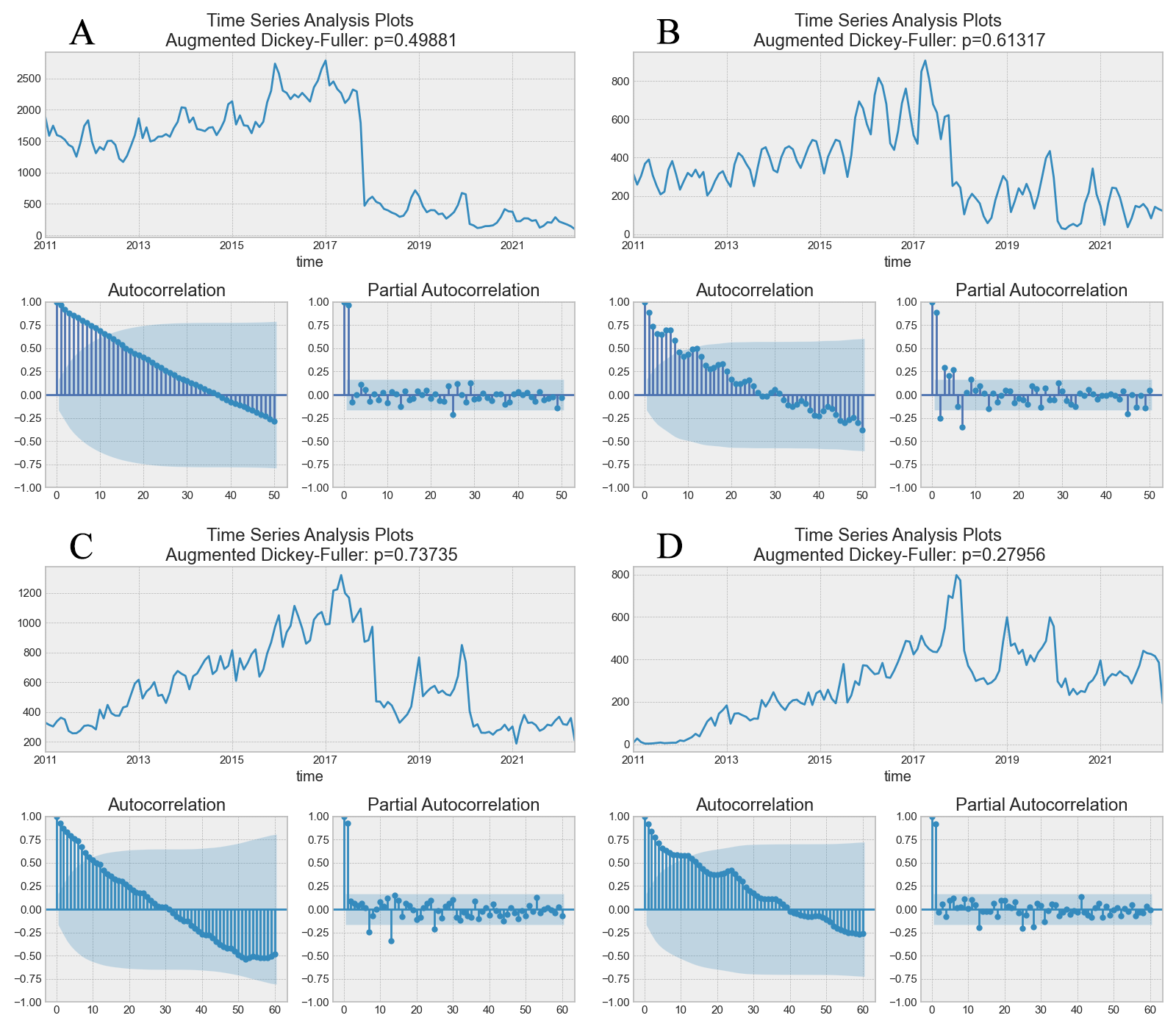


Appendix Figure 8. Augmented Dickey-Fuller test , Auto Correlation Function (ACF) graphs and Partial Auto Correlation Function (PACF) graphs of inpatient. A is 0-1 years old group, B is 2-4 years old group, C is 5-64 years old group, D is ≥65 years old group.


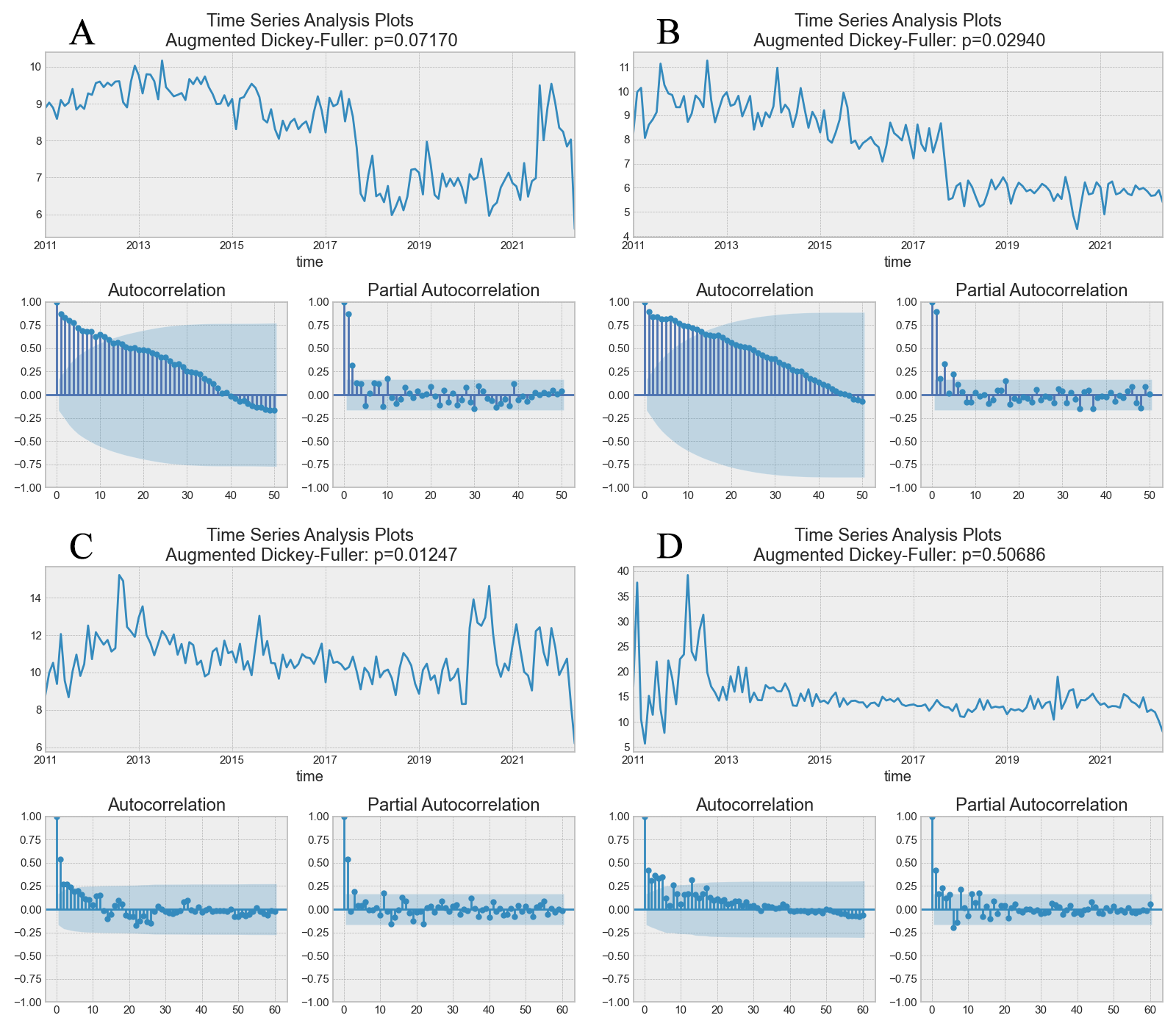


Appendix Figure 9. Augmented Dickey-Fuller test , Auto Correlation Function (ACF) graphs and Partial Auto Correlation Function (PACF) graphs of average length of stay per month. A is 0-1 years old group, B is 2-4 years old group, C is 5-64 years old group, D is ≥65 years old group.


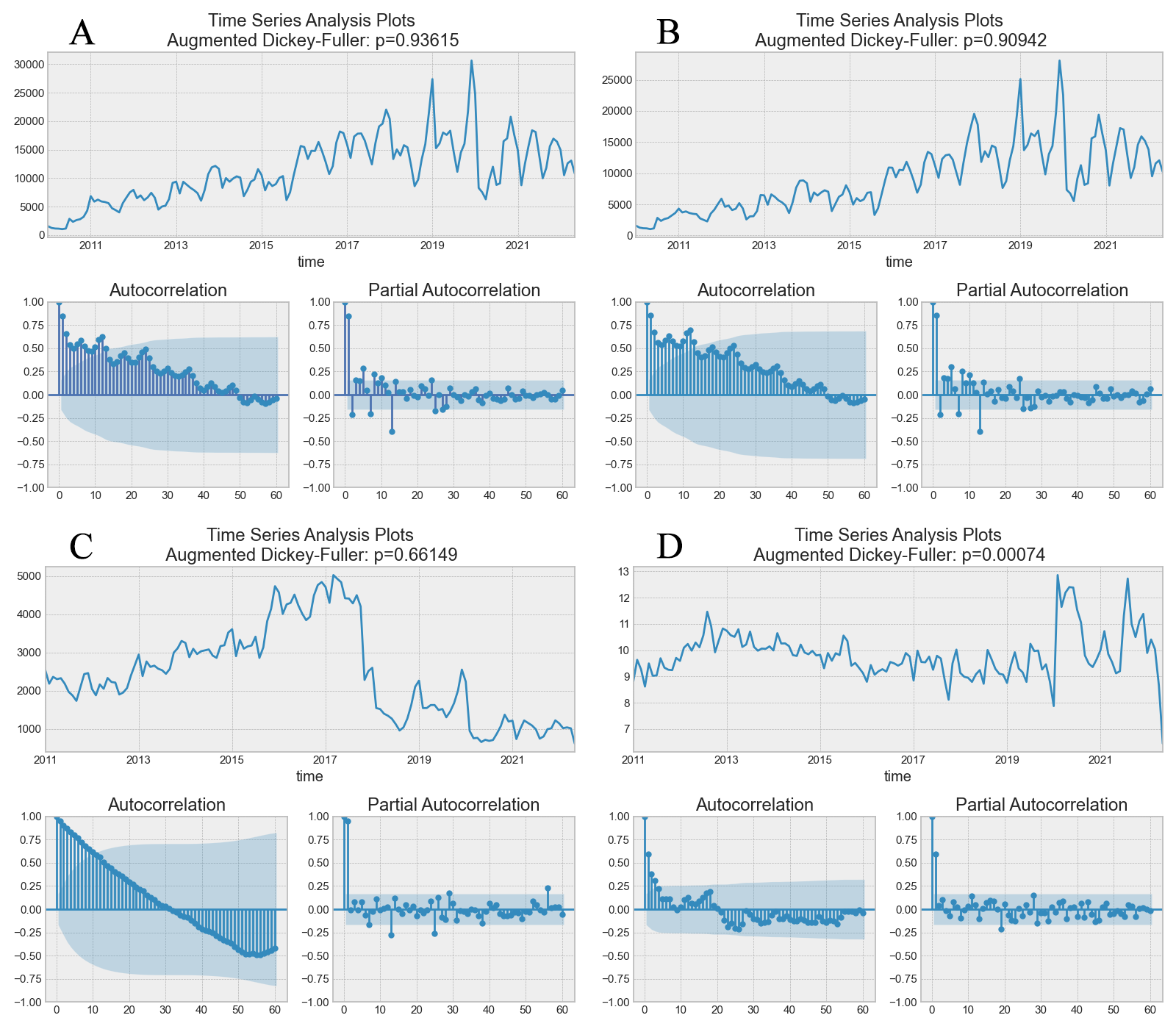


Appendix Figure 10. Augmented Dickey-Fuller test , Auto Correlation Function (ACF) graphs and Partial Auto Correlation Function (PACF) graphs of number of patients by medical visit type and LOS. A is all patients group, B is outpatient and emergency group, C is inpatient group, D is average length of stay per month.


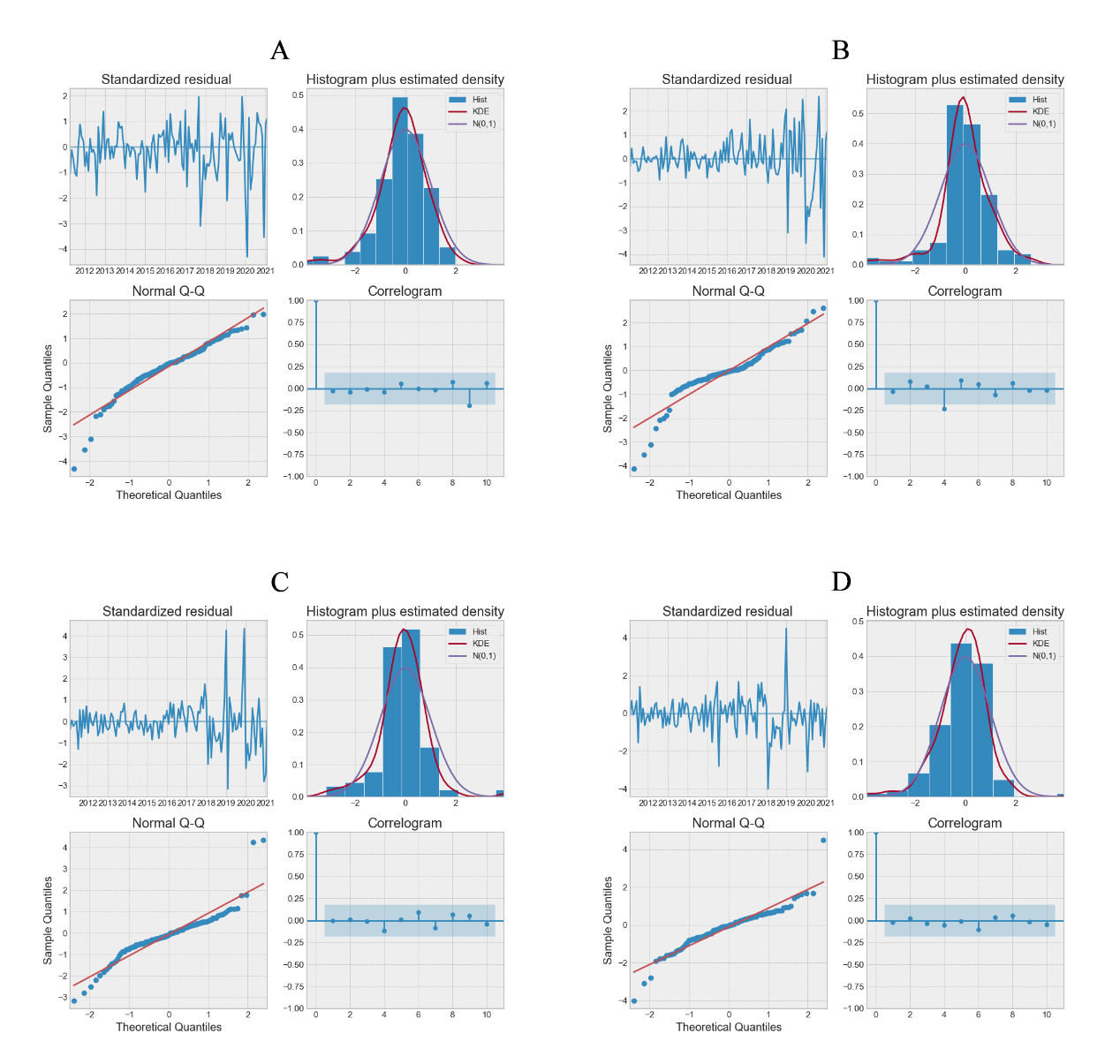


Appendix Figure 11. Optimal SARIMAX model diagnosis of number of patients. A is 0-1 years old group, B is 2-4 years old group, C is 5-64 years old group, D is ≥65 years old group.


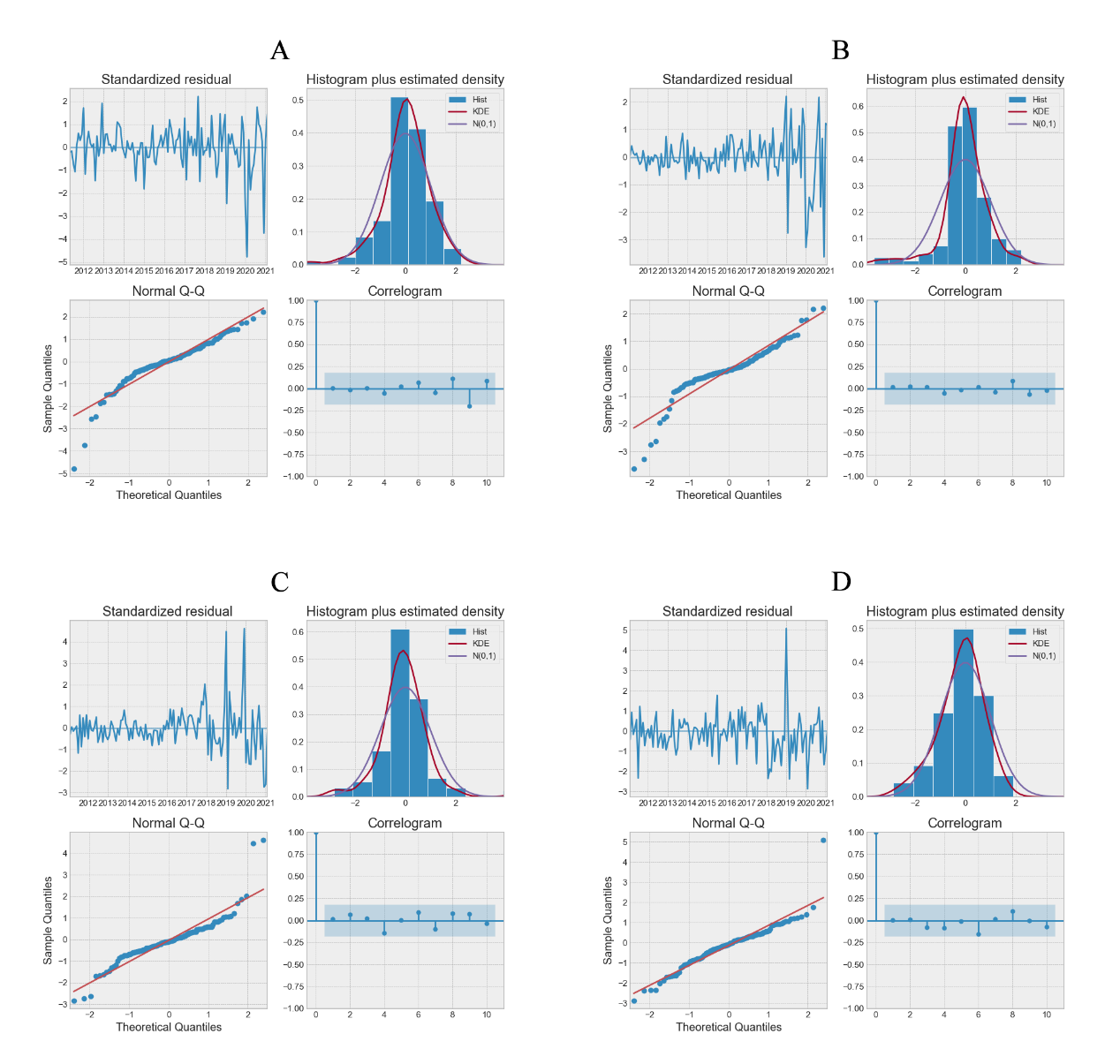


Appendix Figure 12. Optimal SARIMAX model diagnosis of outpatient and emergency. A is 0-1 years old group, B is 2-4 years old group, C is 5-64 years old group, D is ≥65 years old group.


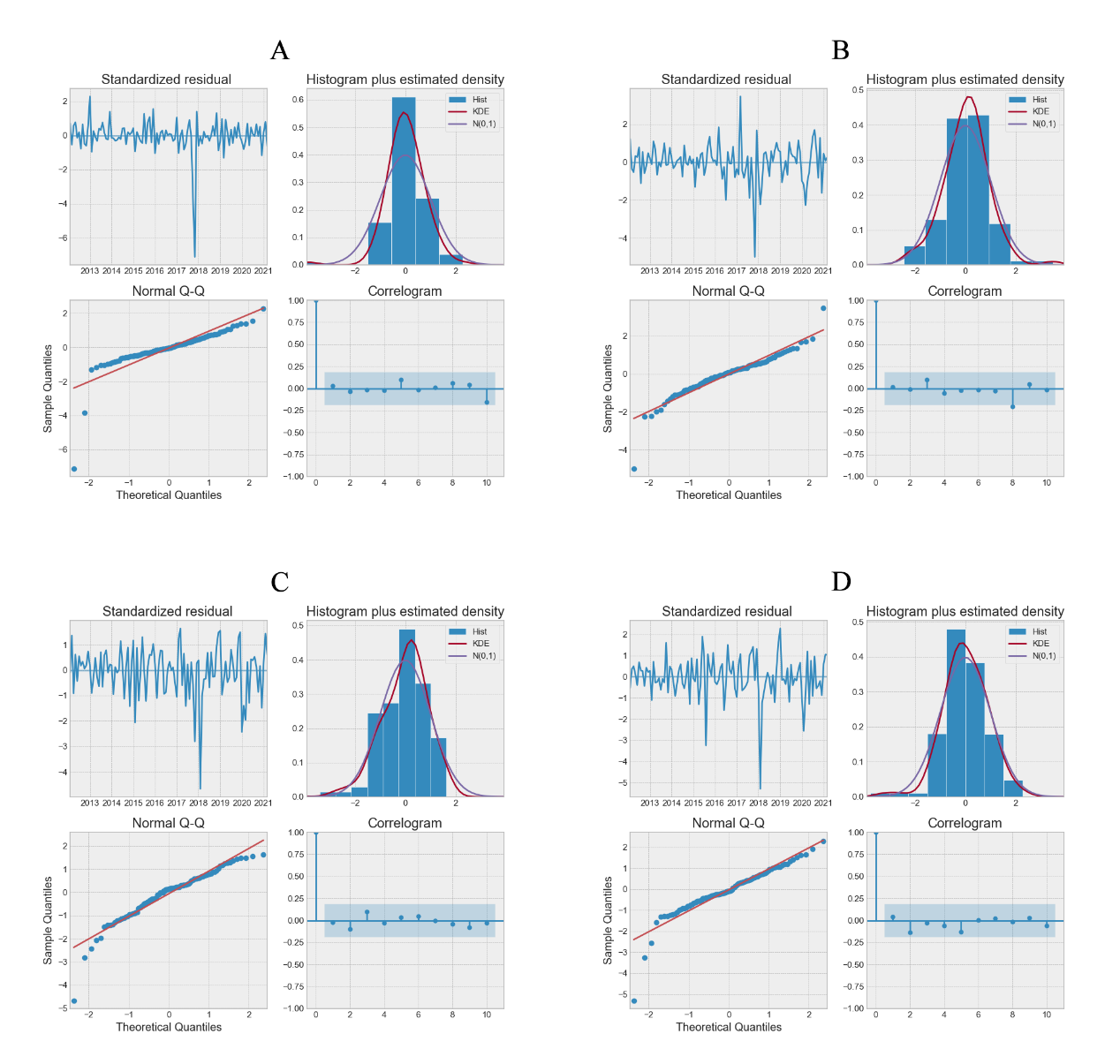


Appendix Figure 13. Optimal SARIMAX model diagnosis of inpatient. A is 0-1 years old group, B is 2-4 years old group, C is 5-64 years old group, D is ≥65 years old group.


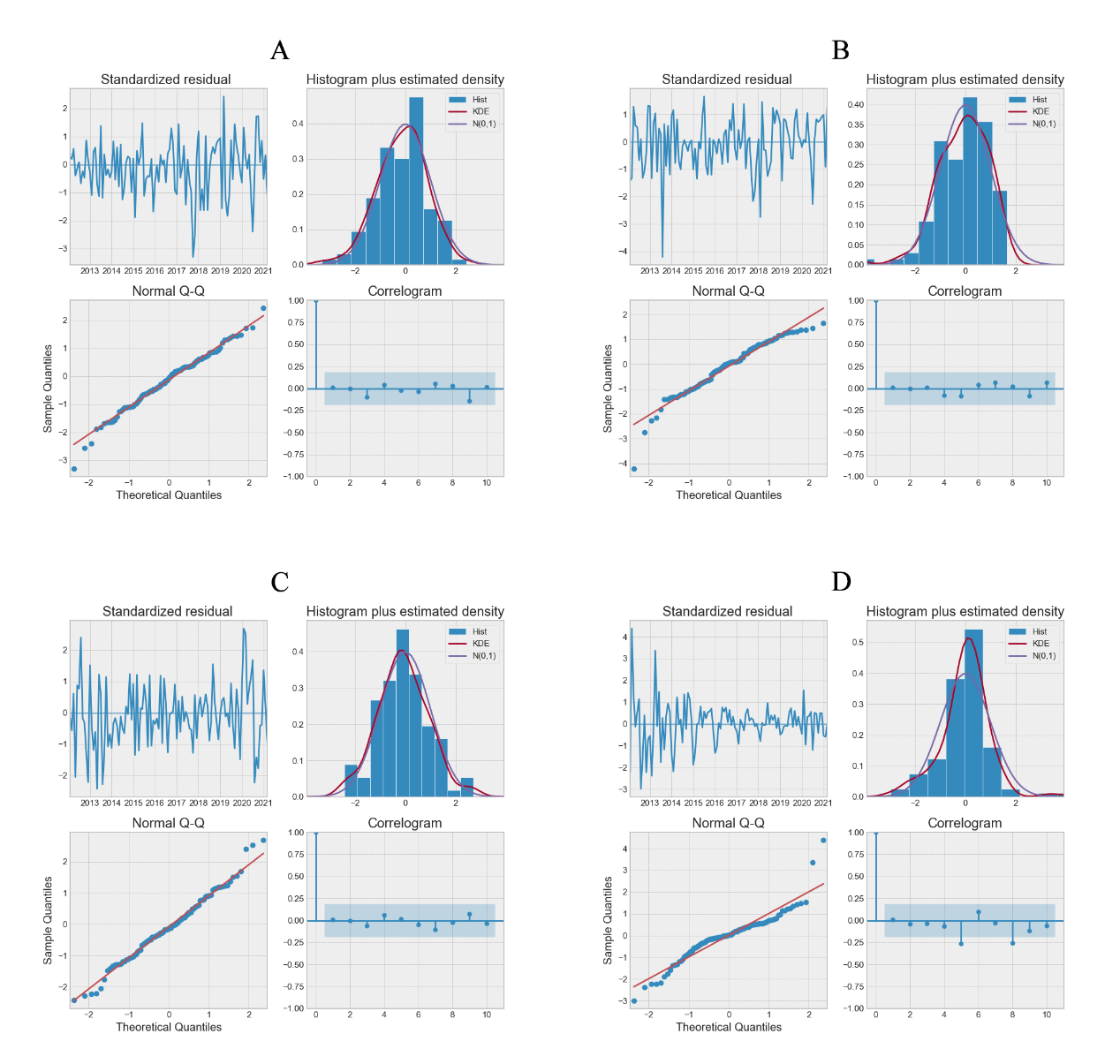


Appendix Figure 14. Optimal SARIMAX model diagnosis of average length of stay per month. A is 0-1 years old group, B is 2-4 years old group, C is 5-64 years old group, D is ≥65 years old group.


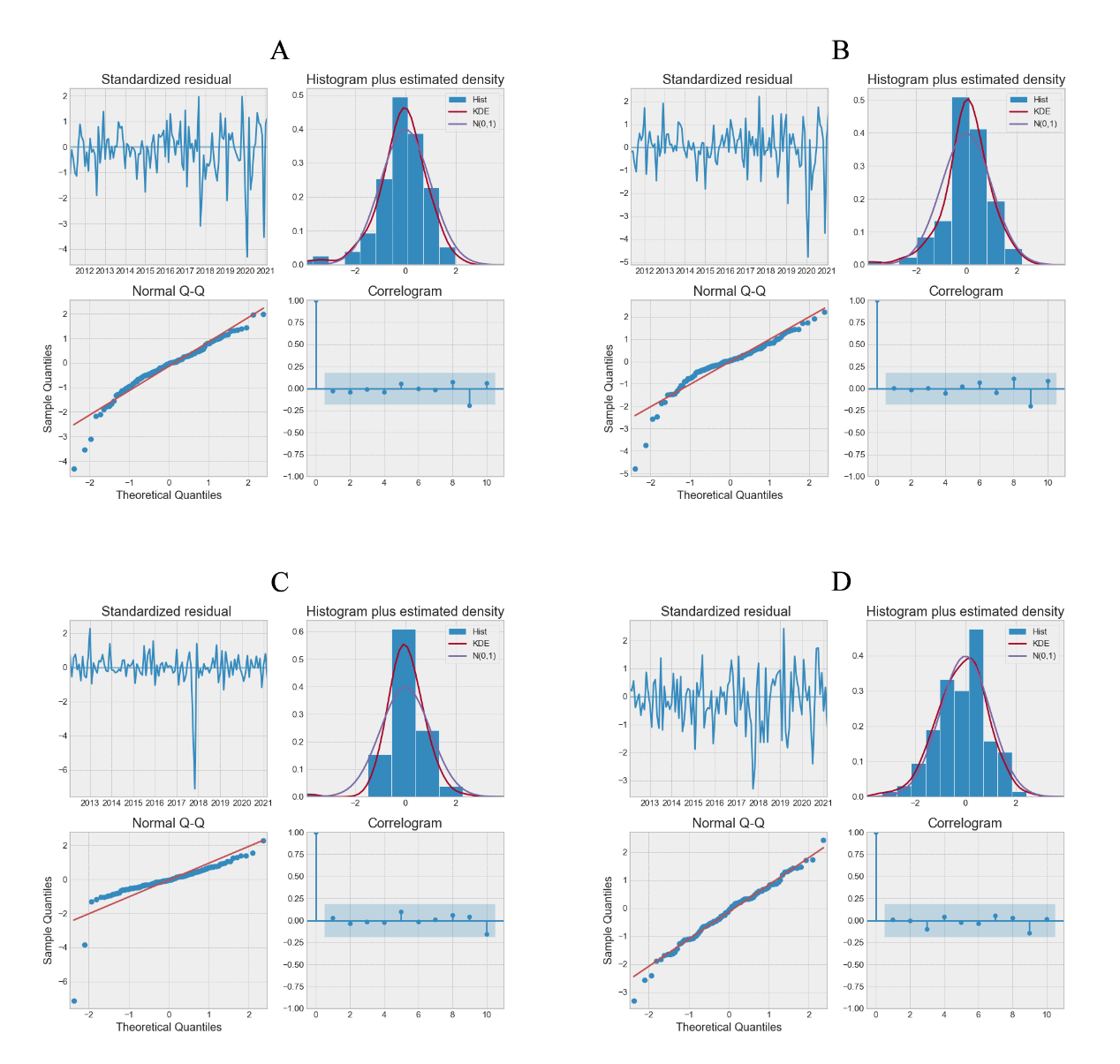


Appendix Figure 15. Optimal SARIMAX model diagnosis of number of patients by medical visit type and LOS. A is all patients group, B is outpatient and emergency group, C is inpatient group, D is average length of stay per month.
